# Gene-specific patterns of expression variation across organs and species

**DOI:** 10.1186/s13059-016-1008-y

**Published:** 2016-07-08

**Authors:** Alessandra Breschi, Sarah Djebali, Jesse Gillis, Dmitri D. Pervouchine, Alex Dobin, Carrie A. Davis, Thomas R. Gingeras, Roderic Guigó

**Affiliations:** Centre for Genomic Regulation (CRG), The Barcelona Institute of Science and Technology, Dr. Aiguader 88, Barcelona, 08003 Spain; Universitat Pompeu Fabra (UPF), Barcelona, Spain; GenPhySE, Université de Toulouse, INRA, INPT, INP-ENVT, Castanet Tolosan, France; Cold Spring Harbor LaboratoryCold Spring Harbor, NY, 11742 USA

**Keywords:** RNA-seq, Comparative transcriptomics, Gene expression, Clustering, Vertebrate, Organ, Species

## Abstract

**Background:**

A comparison of transcriptional profiles derived from different tissues in a given species or among different species assumes that commonalities reflect evolutionarily conserved programs and that differences reflect species or tissue responses to environmental conditions or developmental program staging. Apparently conflicting results have been published regarding whether organ-specific transcriptional patterns dominate over species-specific patterns, or vice versa, making it unclear to what extent the biology of a given organism can be extrapolated to another. These studies have in common that they treat the transcriptomes monolithically, implicitly ignoring that each gene is likely to have a specific pattern of transcriptional variation across organs and species.

**Results:**

We use linear models to quantify this pattern. We find a continuum in the spectrum of expression variation: the expression of some genes varies considerably across species and little across organs, and simply reflects evolutionary distance. At the other extreme are genes whose expression varies considerably across organs and little across species; these genes are much more likely to be associated with diseases than are genes whose expression varies predominantly across species.

**Conclusions:**

Whether transcriptomes, when considered globally, cluster preferentially according to one component or the other may not be a property of the transcriptomes, but rather a consequence of the dominant behavior of a subset of genes. Therefore, the values of the components of the variance of expression for each gene could become a useful resource when planning, interpreting, and extrapolating experimental data from mouse to humans.

**Electronic supplementary material:**

The online version of this article (doi:10.1186/s13059-016-1008-y) contains supplementary material, which is available to authorized users.

## Background

The laboratory mouse has been the top choice organism to model human physiology and disease for decades. The underlying assumption is that the molecular, cellular, and developmental pathways are essentially conserved between human and mouse, and, in general, among placental mammals. The architecture of these pathways is broadly reflected in cellular, tissue, and organ transcriptomes. Therefore, transcriptome comparisons across multiple homologous organs between human and mouse, or across multiple mammalian (or vertebrate) species, have been extensively carried out. Early studies concluded that transcriptional patterns are more similar between homologous organs of different species than between different organs from the same species [1–5], supporting, in principle, the use of the mouse as a model of human biology. Recent results have suggested, however, that these observations may arise from the analysis of a relatively small number of organs that exhibit a disproportionately large number of organ-specific genes. Indeed, by including a larger panel of organs in the analysis, Lin et al. [6] show that transcriptional patterns have overall diverged substantially between human and mouse, separating the species more than the organs. This has led to a highly charged debate [7].

In most cases, the conclusions are essentially of a qualitative nature, obtained after visually inspecting the projection of the transcriptome samples into a space of reduced dimension. Indeed, each transcriptome can be represented as a point in an *n*-dimensional space, its coordinates corresponding to the expression values of *n* genes (in human–mouse comparisons, *n* is typically around 15,000, the number of orthologous protein-coding genes between the two species). Dimensionality reduction is often obtained using principal component analysis (PCA) or related techniques. In PCA, the original values (gene expression levels) are linearly transformed into a set of uncorrelated variables called principal components (PCs). This transformation is defined in such a way that the first PC has the largest possible variance, and each succeeding component has the highest variance possible under the constraint that it is orthogonal to the preceding components. Typically, the two or three first components are chosen and the samples (transcriptomes) are plotted in the corresponding two- or three-dimensional space. The debate is usually centered on whether the samples projected into this space of reduced dimension visually cluster by species [6, 8, 9] or by organ [1–3, 10]. Visual analysis, however, is qualitative in nature, and therefore, has a strong subjective component. To produce, instead, a quantitative criterion, and to avoid, at the same time, the information loss implicit in dimensionality reduction methods, we used here the modularity of the correlation network of the transcriptome samples with respect to the partition of the set of samples, either by organ or by species.

Moreover, the approach above implicitly assumes an average behavior for genes, ignoring that each gene may have a specific pattern of expression variation across organs and species. In fact, we recently showed [11], using transcriptome comparisons of a large collection of human cell lines and mouse organs, that a substantial fraction of genes exhibits constrained expression simultaneously across organs and species within vertebrates. These genes are likely to contribute little to the clustering of transcriptomes in either direction. On the other hand, among the genes whose expression is unconstrained, some may exhibit transcriptional patterns that vary mostly across organs or mostly across species. We previously used linear models to quantify, for each gene, the relative contribution of these two factors (species and organ) to the variation of expression of each individual gene, comparing human and mouse organs [12]. However, since we used only two species, the estimates of variance across species were unreliable. Here, we extend this approach by analyzing previously published transcriptional data in matched samples from six orthologous organs in seven vertebrate species [2]. Using linear models, we quantify, for each gene, the amount of expression variation that originates from variation across organs and from variation across species.

We find that a large fraction of the variance in gene expression (about 70 % on average) can be explained by either organ or species, with the contribution of organ, on average, being larger than that of species. However, we find strong differences between genes in their pattern of expression variation. Genes whose expression varies considerably across species and little across organs lead, as expected, to a species-dominated clustering. These genes exhibit features characteristic of housekeeping genes, and divergence of their expression essentially reflects evolutionary distance. Genes whose expression varies considerably across organs and little across species lead, in contrast, to an organ-dominated clustering. These genes should be specific to a few organs and be essential for their function. Using the projection score [13], we found that a small subset of these genes neatly reproduces the clustering obtained when using all genes. For these genes, animal (and, in particular mouse) models may be particularly appropriate. Interestingly, we found that these genes are much more likely to be associated with diseases than genes whose expression varies considerably across species but little across organs.

## Results and discussion

**Fig. 1:**
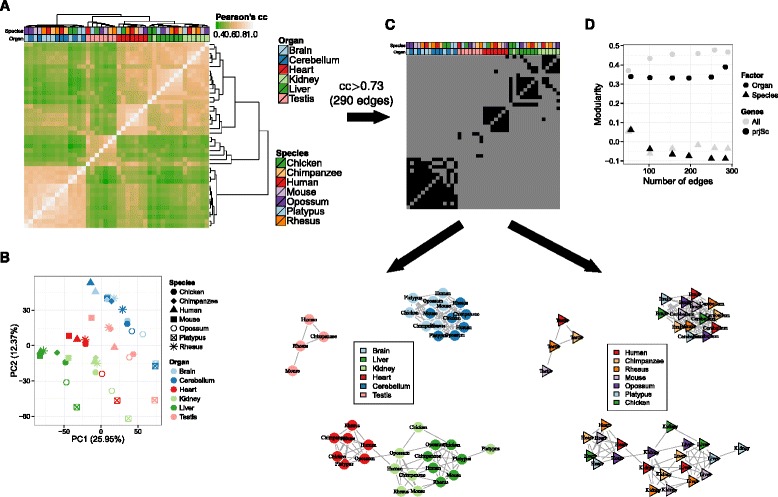
Hierarchical clustering (**a**) and PCA (**b**) based on the expression of 6283 orthologous genes in six organs from seven species show a predominant clustering of organs. Gene expression is computed as log10-normalized cRPKM, with a pseudocount of 0.01. Pearson’s correlation coefficient is computed for each pair of samples. The distance metric used for clustering is again Pearson’s correlation coefficient, and a complete linkage algorithm is applied to the Pearson’s correlation coefficients between each pair of samples. PCA was performed on the same log10-normalized cRPKM, after centering and scaling the expression of each gene across all samples. **c** Example of a network built from the pairwise correlation coefficients (*top*). In such a network (*bottom*), samples are nodes, and edges are drawn when two samples have a correlation coefficient higher than a given threshold (0.73 in the example, which gives 290 edges, as in the last point of **d**). Network nodes are colored either by organ (*left*) or species (*right*), which are the factors used to compute the modularity (see “Methods”). **d** Modularity analysis for the network of gene expression correlations made from six tissues and seven species. The modularity is given as a function of the number of edges in the network, when vertex type is organ (*circle*) or species (triangle), and when the genes considered are all genes (*gray*) or only the projection score genes (*black*). *cc* correlation coefficient, *PC* principal component, *PCA* principal component analysis, *prjSc* projection score

We used gene expression values estimated by RNA-seq in a panel of six organs in seven different vertebrate species from [2]. We restricted the analyses to the set of 6283 protein-coding genes that could be identified as orthologs across the seven species (“Methods”) and used log-transformed expression values, originally computed as cRPKMs, a slightly modified version of the more common RPKM measure, which considers only reads mapping to orthologous genes [2]. Using PCA and hierarchical clustering, we found that if the transcriptomes are considered globally, the samples cluster preferentially by organ (Fig. 1a, b). To quantify the visual interpretation of the clustering/PCA and to overcome the loss of information implicit in this interpretation, we carried out a modularity analysis [14]. Given a network and a grouping of nodes, the modularity measures the degree to which nodes are preferentially connected within the groups (Fig. 1c and “Methods”). Modularity is calculated as the excess number of edges compared to randomly connected nodes, divided by the total number of edges (see “Methods”). In our case, modularity is computed on the network constructed from gene expression correlations between samples when the data is grouped by organ or by species. Grouping by organ yields higher modularity than grouping by species, robustly for any threshold on the correlation defining the network edges (Fig. 1d).

The clustering in Fig. 1a is dominated by the organs, in agreement with published results using transcriptome data on the same or a similar set of organs and species [2, 3]. However, Lin et al. [6] suggested that this organ-dominated clustering is the consequence of the analysis of a limited number of organs, characterized by a large number of organ-specific genes. We re-analyzed Lin’ et al. data using modularity. When restricting to the five organs in common with the dataset in Barbosa-Morais et al. [2] (brain, liver, kidney, heart, and testes), which are the organs with a higher number of organ-specific genes in Lin et al., organ modularity was indeed higher than species modularity (Additional file 1: Figure S1A). However, when using instead the five organs that in Lin et al. have fewer organ-specific genes, species modularity was higher than organ modularity (Additional file 1: Figure S1B), supporting clustering by species. This, indeed, suggests that global transcriptome clustering by organ or species depends on the organs considered (as also recently reported by Sudmant and colleagues [15]). While Lin et al. used a more extensive set of genes (around 15,106 genes), as they only required orthology between human and mouse, the results are comparable to those obtained when using only the vertebrate orthologs of the Barbosa-Morais study [2], even though these genes are likely to be more conserved (Additional file 1: Figure S2A, B).

**Fig. 2:**
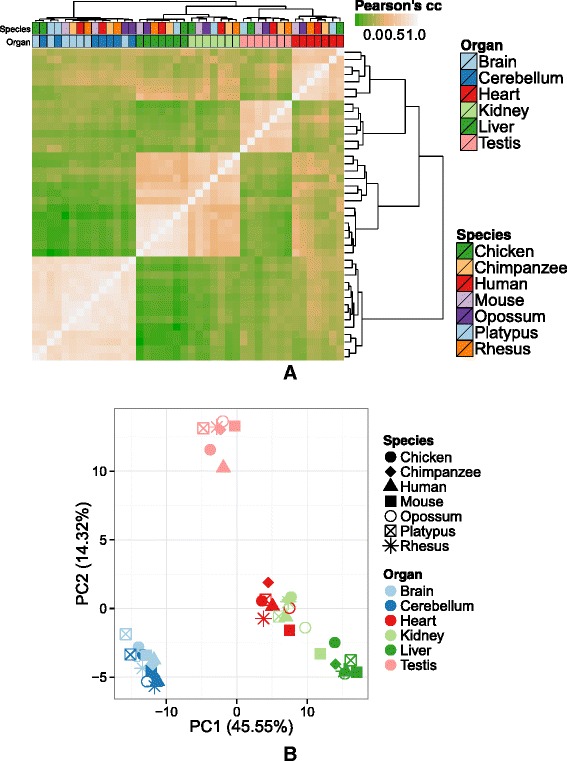
Hierarchical clustering (**a**) and PCA (**b**) based on the expression of 256 genes identified by the projection score [13] show a stronger organ-dominated clustering than when including all genes. Clustering and PCA were performed in the same way as in Fig. 1. *cc* correlation coefficient, *PC* principal component, *PCA* principal component analysis

To identify the set of genes that contribute significantly to the separation between the Barbosa-Morais et al. samples [2], we used the projection score [13] (“Methods”, Additional file 1: Figure S3A). We identified 256 genes that capture most of the variation between samples. PCA and clustering based only on these genes recapitulated very precisely and actually increased the resolution of the results found using the entire set of orthologous genes (Fig. 2a, b), with nearly no change in modularity (Fig. 1d), and allowed us to identify clearly organ-specific genes whose expression is conserved throughout all vertebrates (Additional file 1: Figure S3B). Nearly all these genes have unconstrained gene expression. Indeed, following the criterion in Pervouchine et al. [11], we used a dynamic range minimum threshold of two (i.e., a difference in expression greater than two orders of magnitude) to identify 3622 genes with unconstrained expression across the species and organs in the Barbosa-Morais et al. dataset (Additional file 2: Table S1). These genes include 255 of the 256 genes that drive the clustering of all samples.

To estimate the relative contribution of the variance across organs and across species to the total variance of the expression levels of a given gene, we used linear models (“Methods”). More precisely, we built a linear model for each gene, in which the gene expression level was decomposed into the contribution of the organ, the contribution of the species, and an additional residual error. Thus, as in the ANOVA type of analysis, the total gene expression variance (or total sum of squares, SST) across all observations/samples can be decomposed into three variances: across organs, across species, and the residual variance. The relative contribution of each of these factors to the total gene variance in expression can then be computed as the relative proportion of each variance with respect to the total variance (“Methods”).

**Fig. 3:**
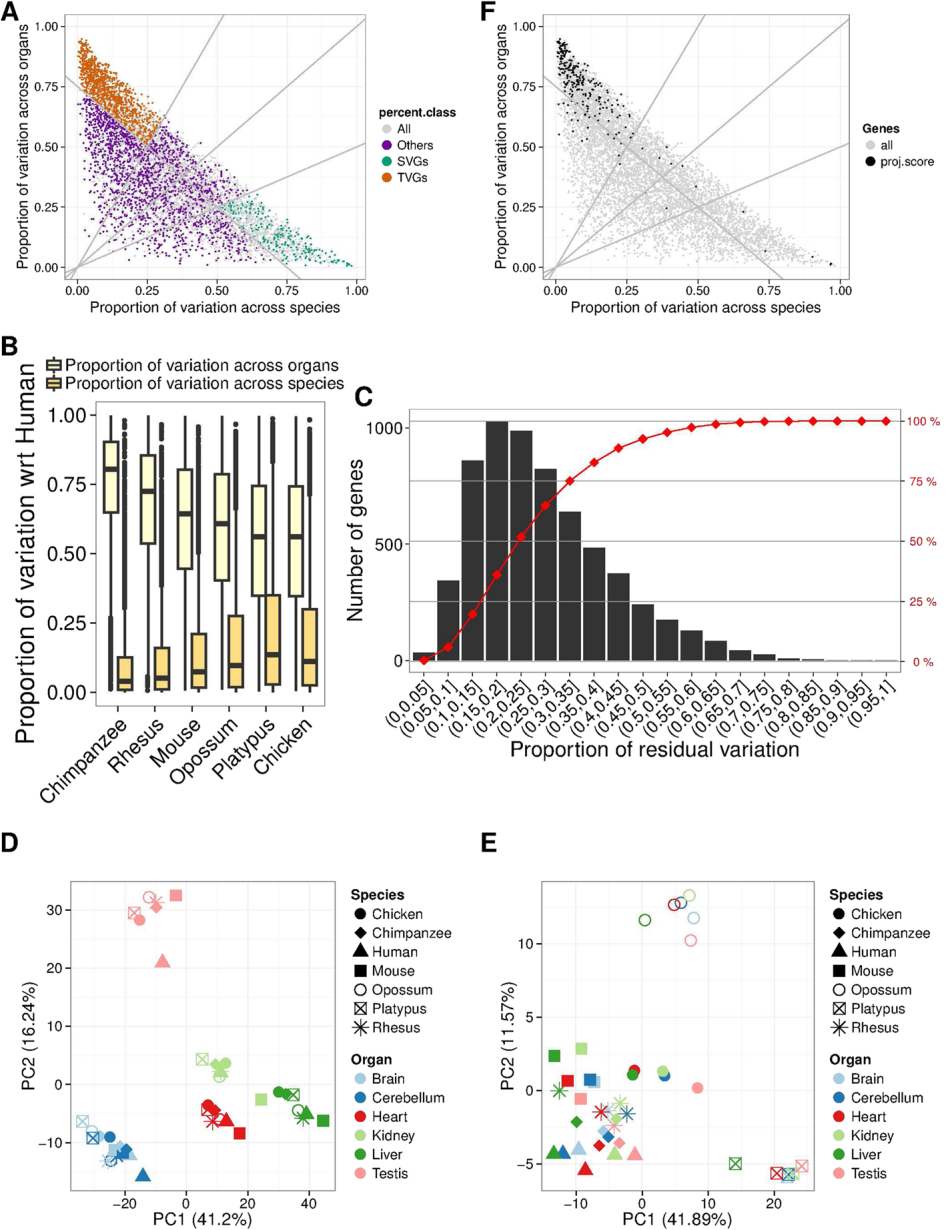
**a** Proportion of expression variance explained by species (*x*-axis) and by organs (*y*-axis) for each of the 6283 orthologous genes (*dots*) using linear models. The *dashed lines* at *y*=2*x* and *x*=2*y* identify the genes in which species-explained variance is twofold greater than organ-explained variance, and in which tissue-explained variance is twofold greater than species-explained variance, respectively. We restricted to the genes for which either species or tissue explains at least 75 % of the variance (*dashed line* at *x*+*y*=0.75), and defined two sets of genes: genes whose expression varies considerably across species and little across tissues, SVGs (*green*), and genes whose expression varies considerably across tissues and little across species, TVGs (*orange*). **b** Box plot representing the distribution of the proportion of gene expression variance across organs (*light yellow*) or between human and each other species (*dark yellow*). When considering more evolutionarily distant species, the proportion of variance across organs decreases, while that between species increases. **c** Number of genes in distinct bins of proportional residual variance (histogram) and cumulative proportion of genes at increasing bins of proportional residual variance. Altogether, 75 % of orthologous genes have less than 35 % residual variance. **d** PCA based on the expression of TVGs only shows an organ-dominated arrangement of the samples in the space defined by the first two PCs. **e** PCA based on the expression of SVGs only shows a species-dominated arrangement of the samples in the space defined by the first two PCs. **f** Same as (**a**), with different color scale, which highlights the higher variance across organs of genes identified by the projection score [13] (*black*), compared to the rest of the genes (*gray*). *PC* principal component, *PCA* principal component analysis, *SVG* species-variable gene, *TVG* tissue-variable gene

The results of the aforementioned variance decomposition of gene expression applied to the data in Barbosa-Morais et al. [2] are shown in Fig. 3a. On average, more than 70 % of the total variance in gene expression can be explained by either organ or species, with the contribution of organ (41 %) being larger than the contribution of species (31 %), consistent with the global organ-dominated clustering. The relative contribution of each factor depends on the evolutionary distance separating the species compared, with the relative contribution of organ decreasing with distance, and that of species increasing (Fig. 3b). Although it is known that gene sequence and gene expression level evolve particularly rapidly in testis [1], the same trend is observed when testis is removed from the analysis (Additional file 1: Figure S4). In 3255 genes (52 %), variance across either organ or species accounted for at least 75 % of the total variance (Fig. 3c). Among these, we identified 1528 genes that vary substantially more across organs than species (defined as having a proportion of organ variance at least twice that of species), and 819 genes that vary substantially more across species than organs (defined as having a proportion of species variance at least twice that of organ). Many genes with a large fraction of the variance explained by either organ or species, however, show little absolute variance (Additional file 1: Figure S5), and therefore, whether the variance is dominated by organ or species is nearly irrelevant. Thus, we intersected them with the set of 3622 unconstrained genes, and identified 1245 unconstrained genes varying preferentially across organs and 268 unconstrained genes varying preferentially across species. We will refer to these genes as tissue-variable genes (TVGs) and species-variable genes (SVGs), respectively. Predictably, the use of TVGs only resulted in an organ-driven clustering (Fig. 3d, Additional file 1: Figure S6A). Including only SVGs resulted, in contrast, in a species-driven clustering (Fig. 3e, Additional file 1: Figure S6B). Modularity analysis quantifies these observations (Additional file 1: Figure S7). Consistent with the larger absolute variance across organs (Additional file 1: Figure S5), most of the 256 genes identified by the projection score method as driving the clustering are TVGs (219, i.e., 86 %), and almost none are SVGs (five, i.e., 2 %, Fig. 3f).

**Fig. 4:**
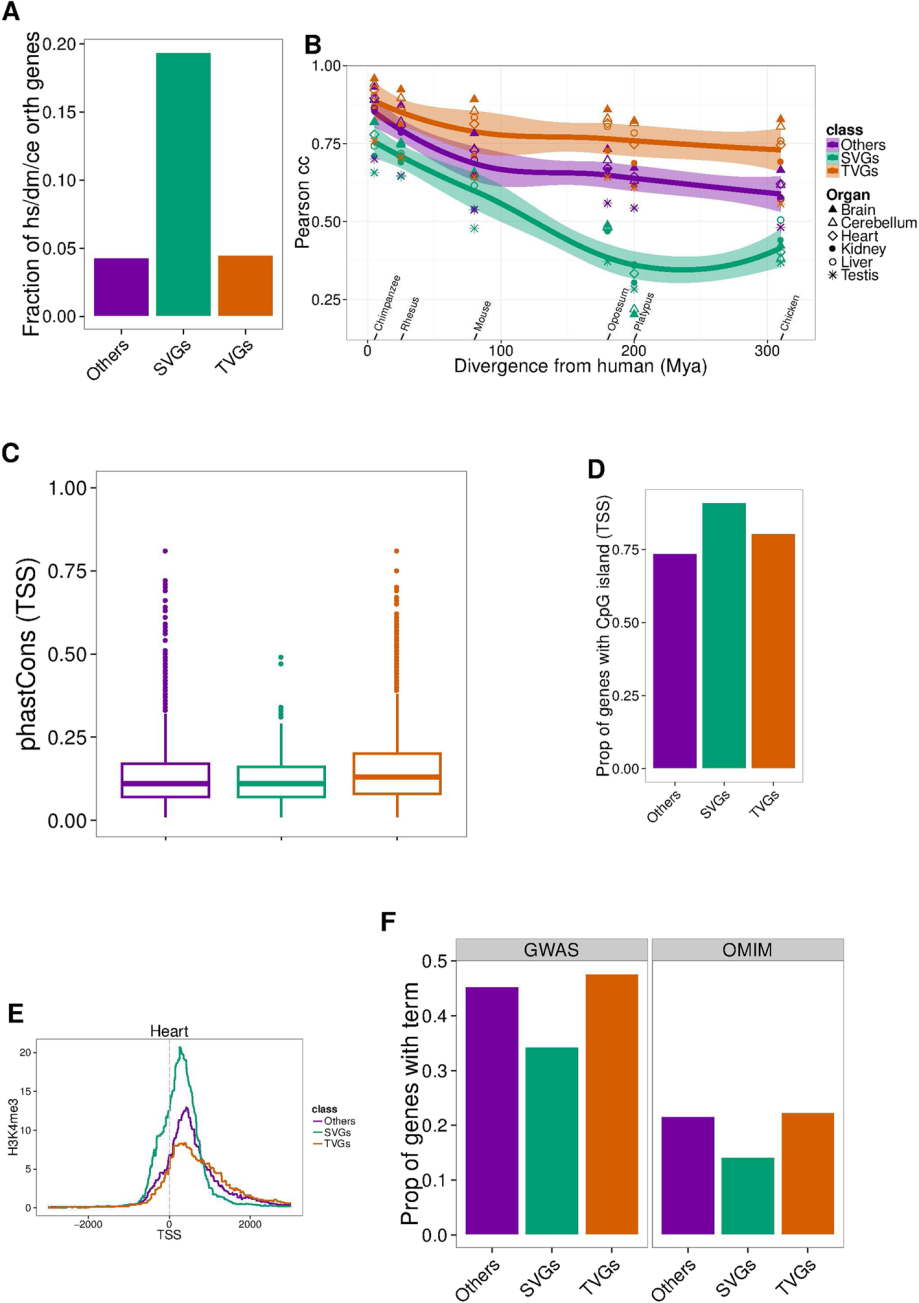
**a** Proportion of genes in each category that have a one-to-one ortholog in human, fly, and worm, as defined by the modENCODE consortium [16]. **b** Pearson’s correlation coefficient between gene expression in each human organ and the corresponding one in every other species. The correlation is computed across all the genes in each class separately. **c** Promoter sequence conservation measured as the average PhastCons signal at TSS (in a window between 3000 bp upstream and 500b p downstream of the TSS). Promoter sequence conservation is higher for TVGs than for SVGs (*p*=4×10^−4^, Mann–Whitney test). Proportion of genes with a promoter category based on CAGE signal [27]. Broad: All the promoters of a gene are broad; sharp: all the promoters of a gene are sharp; mixed: a gene has at least one broad and one sharp promoter; unassigned: none of the promoters of a gene have an assigned category. **d** Proportion of genes in each category covered by CpG islands (as defined in Wu et al. [17]). SVGs have higher CpG island coverage at their promoter than TVGs (*p*=6×10^−5^, chi-squares test). **e** H3K4me3 average signal at TSS (±3000 bp) of a subset of SVGs, heart-specific TVGs, and others. Genes in each category are filtered to have comparable levels of expression (“Methods”). **f** Proportion of genes in each category with an associated GWAS trait or OMIM disease. *cc* correlation coefficient, *SVG* species-variable gene, *TSS*, transcription start site, *TVG* tissue-variable gene

In general, SVGs exhibit properties characteristic of housekeeping genes, and gene ontology (GO) analysis does indeed indicate that they are involved in basic cellular functions (Additional file 1: Figure S8), compared to TVGs. As expected, SVGs are evolutionarily older than TVGs, since 19 % of them are present across all metazoans [16], compared to only 4 % of TVGs (Fig. 4a, “Methods”). In SVGs, divergence in gene expression is almost directly related to evolutionary divergence. Indeed, we computed the Pearson’s pairwise correlation of expression across genes between human and each other species for each organ separately, as in Barbosa-Morais et al. [2]. We observed a strong dependence and steep decline when increasing the evolutionary distance for SVGs, which was very moderate for TVGs (Fig. 4b).

We also found that promoters of TVGs show stronger sequence conservation than those of SVGs (*p*=4×10^−4^, Mann–Whitney test, Fig. 4c, “Methods”), and that they tend to overlap CpG islands less frequently [17] (*p*=6×10^−5^, chi-squares test, Fig. 4d, “Methods”). It has been shown that promoters of housekeeping genes are associated with higher CpG island overlap [18]. On the other hand, TVGs show a weaker H3K4me3 signal, a histone modification typical of transcription initiation (as measured by the ENCODE Project in five mouse organs [12]). Instead, SVGs are enriched in this mark, compared to TVGs (*p*(Heart)=2×10^−2^, Mann–Whitney test, Fig. 4e, Additional file 1: Figure S9), even for a subset of genes with comparable expression levels (“Methods”). Again, this difference has been observed between the promoters of housekeeping genes and tissue-specific genes (see, for instance, [19]).

It is sensible to assume that animal models will be particularly appropriate for genes whose expression varies considerably across organs, but little across species (TVGs). Interestingly, we found that TVGs are more likely to be associated with diseases (as reported in OMIM [20] and the GWAS catalogue [21]), than SVGs (Fig. 4f).

Our results overall show that meaningful organ- and species-dominated transcriptome clustering can be obtained by selectively considering genes with high variation across organs and little across species, and vice versa. Ultimately, the clustering will be dominated by the factor (organ or species) that dominates the variance. A similar outcome can be produced, therefore, on the whole set of orthologous genes by employing gene expression normalization methods that shift the variance in one direction or another. Most dramatically, if we scale the expression of each gene across organs (species), the variance of expression across organs (species) would be canceled out. Consequently, TVGs increase their relative variance contribution by species when scaling across organs, and SVGs increase their relative variance contribution by organ when scaling across species (Fig. ??a). When performing PCA on all genes after normalization, transcriptomes consistently cluster by species or organ depending on whether scaling of gene expression has been performed by organ or by species (Fig. ??b, c).

**Fig. 5:**
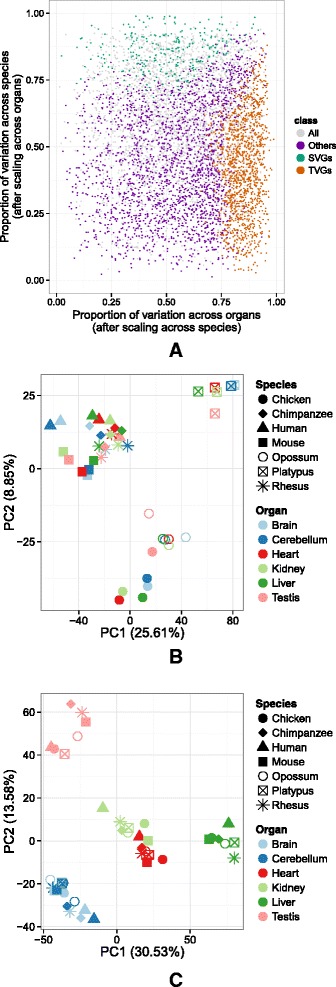
**a** Proportion of expression variance explained after centering and scaling each gene expression across species (*x*-axis) or across organs (*y*-axis) for the 6283 orthologous genes. When centering and scaling across species, the variance explained by species is 0 and there is only variance explained by organ (*x*-axis). Conversely, the *y*-axis is the proportion of variance explained by species after the variance across organs becomes 0 because of centering and scaling across organs. *Dots* are colored based on the class assigned to each gene. A PCA is performed on the gene expression of all 6283 orthologous genes after centering and scaling their expression across organs (**b**) or across species (**c**). The first PCA shows a species-dominated clustering, while the second one shows an organ-dominated clustering. *PC* principal component, *PCA* principal component analysis, *SVG* species-variable gene, *TVG* tissue-variable gene

Our model does not take into account inter-individual gene expression variation within a given species. However, single measurements in genes whose expression varies considerably across individuals in a given species are not informative of the expression of the gene in that species. Thus, when compared across multiple tissues among species, they may appear to exhibit a stochastic behavior, and could potentially contribute to residual variation. To assess the impact of inter-individual variation in our results, we used gene expression data produced by the GTEx consortium [22] in multiple tissues from multiple post-mortem donors. In the work by Melé et al. [23], we had previously estimated that the average contribution of inter-individual variance to the global variance of gene expression was on average very low (5 %), and here we have found that it is only slightly higher in SVGs than TVGs (Additional file 1: Figure S10A). Because the estimates of the variance decomposition in Melé et al. are inferred from a larger set of tissues than those available in Barbosa-Morais et al. [2], we performed the variance decomposition only on the organs common with the Barbosa-Morais et al. study, and found that in these organs, inter-individual variance was even lower (4 % on average, Additional file 1: Figure S10B). These results suggest that inter-individual variation has little impact on our estimates of inter-organ and inter-species variation.

## Conclusions

Transcriptome comparisons reveal to what extent the biology of a given organism can be extrapolated to another. Regarding specifically human and mouse, intense debate exists as to whether organ transcriptomes, when taken globally, cluster preferentially by organ or by species. This is central to the use of mouse as a model of human biology. Here we used a modularity analysis to measure quantitatively such a preference, beyond the mere visual inspection of the output of dimensionality-reduction techniques typically used to address this question. We specifically used modularity to analyze the results in Lin et al. [6]. These have been challenged on the basis of a potentially flawed experimental design [7]. Human organ samples and mouse organ samples in the initial study by Lin et al. were sequenced in two different batches, making it, indeed, impossible to separate the effect of the batch from that of the species. However, our analysis of the modularity of the correlation networks indicates that the batch effect is unlikely to be the dominant factor, because when we restrict the analysis of the Lin et al. data to the five organs common to the Barbosa-Morais et al. study [2], the clustering observed is by organ and not by species.

It is not our main aim here, however, to take a position on whether human and mouse transcriptomes are preferentially conserved across organs or species, but rather to address the limitations of an approach based on global transcriptome comparisons. This implicitly assumes an average behavior for genes, ignoring that each gene has a characteristic pattern of expression variation across species and organs. Our results show, indeed, that there is continuum in the spectrum of expression variation, at one extreme of which are genes whose expression varies considerably across species and little across organs (and, therefore, lead to a species-dominated clustering), and at the other extreme of which are genes whose expression varies considerably across organs and little across species (and lead, therefore, to an organ-dominated clustering). Therefore, whether transcriptomes, when considered globally, cluster preferentially according to one component or the other, may not be as much a generic property of the transcriptomes, but rather a consequence of the dominant behavior of a subset of genes. Our results actually suggest that the organ-dominated clustering obtained using the Barbosa-Morais et al. whole transcriptome dataset may actually be driven by a small subset of genes whose expression varies largely across organs, and little across species (Additional file 1: Figure S3B).

To assess the impact of inter-individual variation in our estimates of inter-organ and inter-species variation, we have used gene expression data from multiple tissues from multiple human donors. Unfortunately, such data do not exist for other species, which has prevented us from using a more general approach, in which tissues, species, and individuals within species are considered as factors in the linear models.

We believe that by investigating the patterns of expression variation across species and tissues specifically for each gene, we can provide a more meaningful answer to the question of whether the biology of an organism can be extrapolated to another. Indeed, the behavior of genes whose expression is variable across organs but stable across species (that is, the genes that exhibit similar patterns of expression variation across organs in different species) may be more confidently extrapolated across species than that of genes whose pattern of expression variation differs substantially between species. Interestingly, those genes with conserved expression patterns across vertebrate species (and for which, therefore, the mouse may be a good model of human biology) are more often associated with diseases than genes with non-conserved expression patterns across species. We believe, therefore, that the values of the components of the expression variance that we have attached to each gene could become a useful resource when planning, interpreting, and extrapolating experimental data in mouse and other vertebrate model organisms to human.

## Methods

### RNA-seq sample clustering based on gene expression

#### Gene expression matrix

Raw cRPKM values were obtained from the study by Barbosa-Morais et al. [2]. To have a balanced design, the original matrix was restricted to species for which the same six organs were available (see below). The final matrix consisted of seven vertebrates, including human, chimpanzee, rhesus, mouse, opossum, platypus, and chicken, and six organs, including brain, cerebellum, heart, liver, kidney, and testes.

We restricted the analyses to protein-coding genes with a one-to-one orthology relationship in the seven species. We used the orthology relationships of the Barbosa-Morais et al. study [2], which include 6787 orthologous genes. Of these, we retained 6393 orthologs after checking for consistency against each annotation set in Ensembl v65, for each species (genome and annotation files from the Barbosa-Morais study can be found in Additional file 3: Table S2). Finally, we intersected this set with the list of one-to-one protein-coding orthologs between human and mouse provided by the mouse ENCODE consortium [12], to get a final matrix consisting of expression values for 6283 genes in 42 samples (Additional file 3: Tables S3 and S4).

#### Hierarchical clustering and PCA

In Figs. 1a and 2a, the samples are clustered hierarchically based on their pairwise Pearson’s correlation coefficients of gene expression values, where cRPKM are log10-normalized after adding a pseudocount of 0.01. The samples are then clustered on the vector of the correlation coefficients, with one minus Pearson’s correlation coefficient (1−|*r*|) as a distance metric, using the complete linkage clustering algorithm.

In Additional file 1: Figure S6A, B, the samples and genes are clustered hierarchically based on gene expression values directly. Again cRPKM are log10-normalized after adding a pseudocount of 0.01 and the complete linkage clustering algorithm is applied on Euclidean distances.

PCA, as shown in Figs. 1b, 2b and 3d, e, was performed on cRPKM values normalized in the same way, but centered and scaled across all the samples for each gene. PCA, as shown in Fig. ??b, c, however, was performed after centering and scaling the normalized cRPKM for each gene across all the organs in a given species (Fig. ??b), and across all the species for a given organ (Fig. ??c), respectively.

#### Network modularity

The modularity of a graph with respect to some division (or vertex types) measures how good the division is, or how separated the different vertex types are from each other. In this study, we build a graph where samples are vertices (or nodes). Two vertices or samples are connected if the Pearson’s correlation coefficient between them, computed on the gene expression values, is higher than a certain threshold (excluding connections of a sample with itself). As in hierarchical clustering and PCA, gene expression values are log10-transformed cRPKM after adding a pseudocount of 0.01. The vertex types on which the modularity is computed are either the organ or the species classification. To compute the modularity, we used the function modularity() from the R package igraph v0.7.1, which implements the following definition [14]:

1 $$ Q = \frac{1}{2m} \times \sum_{i} \sum_{j} \left[ \left(A_{ij} - \frac{k_{i} \times k_{j}}{2m} \right) \delta(c_{i},c_{j}) \right],  $$

where *m* is the number of edges, *A*_*ij*_ is the element of the adjacency matrix *A* in row *i* and column *j* (corresponding to vertices *i* and *j*, respectively), *k*_*i*_ is the degree of *i*, *k*_*j*_ is the degree of *j*, *c*_*i*_ is the type (or component) of *i*, *c*_*j*_ that of *j*, the sum goes over all *i* and *j* pairs of vertices, and *δ*(*x,y*)=1 if *x*=*y*, and *δ*(*x,y*)=0 otherwise.

Finally, the modularity is plotted as a function of the network density, which is defined as the actual number of edges (based on the threshold of the correlation coefficient) over the total number of possible edges. We set self-connection to 0 in the adjacency matrix even though samples share an identity with themselves, to ensure self-connection does not inflate the modularity calculation. Conclusions are robust to setting self-connection to 1.

### Projection score

The projection score is a measure of the informativeness of a subset of variables with respect to PCA visualization [13]. Here, we subset the variables, i.e., the genes, based on increasing thresholds of their variance across all samples (as a ratio to the maximum variance). For each subset of genes, the projection score is computed over 100 permutations with respect to the first three PCs (Additional file 1: Figure S3A), and the subset with the highest score is selected for further analyses. This subset includes 256 genes (Additional file 3: Table S5), and their log10-transformed cRPKM values are shown in Additional file 1: Figure S3B.

#### Linear models, variance decomposition, and SVG and TVG definition

The expression of each gene in a given sample is usually dependent on the identity of the sample, which here is represented by the organ and the species of origin. More formally, for an individual gene, a linear model can be built that describes its expression as the sum of the factors organ and species and a residual term:

2 $$ y_{ij} = \mu + \text{org}_{i} + \text{spc}_{j} + \epsilon_{ij},  $$

where *y*_*ij*_ is the expression of a gene in organ *i* (of *n*_o_ organs) and species *j* (of *n*_s_ species), *μ* is the basal expression level of the gene, org_*i*_ is the coefficient for organ *i*, spc_*j*_ is the coefficient for species *j*, and *ε*_*ij*_ is the residual term.

Thus, as in the ANOVA type of analysis, the total gene expression variation for each gene (or total sum of squares, SST_g_) across all samples can be decomposed into three variations: variation across organs (SSO_g_), variation across species (SSS_g_), and a residual variation (SSR_g_):

3 $$ \text{SST}_{\mathrm{g}} = \text{SSO}_{\mathrm{g}} + \text{SSS}_{\mathrm{g}} + \text{SSR}_{\mathrm{g}},  $$

where

4 $$ \text{SST}_{\mathrm{g}} = \sum\limits_{i=1}^{n_{\mathrm{o}}}\sum\limits_{j=1}^{n_{\mathrm{s}}}(y_{ij}-\bar{y}_{\cdot\cdot})^{2},  $$

5 $$ \text{SSO}_{\mathrm{g}} = n_{\mathrm{s}}\sum\limits_{i=1}^{n_{\mathrm{o}}}(\bar{y}_{i{\cdot}} - \bar{y}_{\cdot\cdot})^{2},  $$

6 $$ \text{SSS}_{\mathrm{g}}=n_{\mathrm{o}}\sum\limits_{j=1}^{n_{\mathrm{s}}}(\bar{y}_{{\cdot}j} - \bar{y}_{\cdot\cdot})^{2},  $$

7 $$ \text{SSR}_{\mathrm{g}}=\sum\limits_{i=1}^{n_{\mathrm{o}}}\sum\limits_{j=1}^{n_{\mathrm{s}}}(y_{ij} - \bar{y}_{i\cdot} - \bar{y}_{{\cdot}j} + \bar{y}_{\cdot\cdot})^{2},  $$

and

8 $$ \bar{y}_{\cdot\cdot}=\sum\limits_{i=1}^{n_{\mathrm{o}}}\sum\limits_{j=1}^{n_{\mathrm{s}}} y_{ij},  $$

9 $$ \bar{y}_{i{\cdot}}=\frac{1}{n_{\mathrm{s}}} \sum\limits_{j=1}^{n_{\mathrm{s}}} {y_{ij}},  $$

10 $$ \bar{y}_{{\cdot}j}=\frac{1}{n_{\mathrm{o}}} \sum\limits_{i=1}^{n_{\mathrm{o}}} {y_{ij}}.  $$

The relative contribution of each factor to the total gene expression variation can then be computed as the relative proportion of each variation with respect to the total. The linear model was implemented using the function lm() from basic R. A convenient in-house wrapper is available at https://github.com/abreschi/Rscripts/blob/master/anova.R.

In a two-factor linear mixed model, the factors organ and species can be considered as giving an independent additive contribution to the gene expression level with variances $\sigma _{\mathrm {o}}^{2}$ and $\sigma _{\mathrm {s}}^{2}$, respectively, along with an independent additive contribution of the residual term that has variance $\sigma _{\mathrm {e}}^{2}$. In this case, the relative contribution of each factor (e.g., organ) to the gene expression variation can be thought of as the variance of that factor over the sum of the variances of both factors plus the residual variance (e.g., $\sigma _{\mathrm {o}}^{2}/(\sigma _{\mathrm {o}}^{2} + \sigma _{\mathrm {s}}^{2} + \sigma _{\mathrm {e}}^{2})$ [23]). The linear mixed models were implemented by using the function lmer() of the R package lme4 v1.1-7.

As the correlation between the relative contributions with the linear model and with the linear mixed model is very high for both factors (Additional file 1: Figure S11A, B), we decided to use the linear model, which requires no estimation step and is more intuitive.

To remove genes with relatively low variability of expression, we filtered them based on their dynamic range, computed on cRPKM after adding a pseudocount of 0.01. The dynamic range for each gene is defined as the difference in order of magnitudes between the maximum and the minimum expression across all samples. We used a minimum threshold of 2 orders of magnitude [11], to retain only the most variable genes, which we refer to as unconstrained. Within this set of unconstrained genes, we further considered genes for which either species or organ explains at least 75 % of the variance (dashed line at *x*+*y*=0.75 on Fig. 3a and f), and defined two sets of genes: genes whose relative variation of expression is twofold greater across species than across organs (SVGs) and genes whose relative variation of expression is twofold greater across organs than across species (TVGs). The unconstrained genes that are neither SVGs nor TVGs are referred to as others.

To find the distribution of the proportion of expression variation between human and each other species (Fig. 3b), we built a linear model for all the organs of human and the other species. The gene expression values were log10-normalized, after adding a pseudocount of 0.01, and centered and scaled within each sample. Since gene expression is known to evolve much faster in testis [1], we performed the same analysis excluding testis. We found the same result (Additional file 1: Figure S4).

### Properties of SVGs and TVGs

#### GO analysis

The GO term enrichment analysis in Additional file 1: Figure S8 was performed separately for each set of genes, with respect to all 6283 orthologous genes in the matrix, used as background. The enrichment is tested with the hypergeometric test implemented in the R package GOstats v2.34.0. Ensembl gene IDs are converted to entrez gene IDs via the R package org.Hs.eg.db v3.1.2, and mapped to gene ontology through the R package GO.db v3.1.2. The GO terms associated with the biological process hierarchy are sorted by their *p* values corrected for multiple testing (Benjamini–Hochberg correction [24]), and the top ten significantly enriched terms are shown for each group of genes.

#### Evolutionarily conserved genes

We computed the fraction of evolutionarily conserved genes as the proportion of genes in each class that were identified as being orthologous between human, fly (*Drosophila melanogaster*), and worm (*Caenorhabditis elegans*) as defined by the modENCODE consortium [16].

#### Promoter analysis

##### Promoter sequence conservation.

The promoter sequence conservation was computed for a window of 2000 bp upstream and 500 bp downstream of the transcription start site (TSS) of each gene. A gene TSS is defined as the most 5^′^ base of the gene. PhastCons scores [25] in this window are averaged from the bigwig file with the bwtool software [26] and this average is taken as a measure of promoter sequence conservation.

##### CpG island coverage.

For each base in a 6000-bp window around the TSS (±3000 bp) of the human genes of our set of one-to-one orthologs, we computed a binary overlap with CpG islands, as annotated in [17]. We then computed the proportion of genes in each class with at least one overlapping CpG island, as shown in Fig. 4d.

##### H3K4me3 signal.

We compared the intensity of the H3K4me3 mark at the promoter of each category of gene in the five organs for which ChIP-seq experiments were available from the mouse ENCODE consortium [12] (Additional file 3: Table S6), namely cerebellum, heart, kidney, liver, and testes. As H3K4me3 is a mark known to be present at the promoter of the majority of actively transcribed genes, we restricted our comparison to a subset of TVGs specific to each organ, and to a subset of SVGs and others that are comparable to this subset of TVGs in terms of number of genes and expression values.

To select genes specific to each organ, we required the genes to be common to multiple species and shared by a limited number of organs. As shown in Additional file 1: Figure S12, five is the number of species for which we have the maximum number of genes specific to one organ and present in this number of species. We identified 1086 such genes (Additional file 1: Figure S13).

For each subset of TVGs specific to a given organ, we selected a subset of SVGs and other genes with the same number of genes and a similar expression. To select genes with comparable expression, we binned the expression values of SVGs, other genes, and TVGs specific to one organ in 50 expression bins. Then, for each bin, we randomly selected a number of genes from each class, corresponding to the minimum number of genes available in that bin.

The average signal intensity for each mark was computed around the TSS (±3000 bp) at each 10-bp bin for the three classes of genes in each organ (Fig. 4e and Additional file 1: Figure S9).

#### GWAS and OMIM analyses

For each category of genes, we computed the proportion of genes with an associated disease in the OMIM database (http://omim.org/, version updated to June 2014) or a trait in the GWAS catalog (https://www.ebi.ac.uk/gwas/, version updated to June 2014). For the genes associated with a GWAS trait, we used the gene reported in the catalog, when available.

### Analysis of inter-individual variation in GTEx

Gene expression values (RPKM) for the latest public GTEx release were downloaded from the GTEx portal (http://www.gtexportal.org/home/datasets/, file: GTEx_Analysis_v6_RNA-seq_RNA-SeQCv1.1.8_gene_rpkm.gct.gz). To apply linear models to a balanced design matrix with organs and individuals, we retained gene expression data from the only four donors for which most of the organs in Barbosa-Morais et al. [2] were available (cerebellum, heart, kidney, liver, and testis; Additional file 3: Table S7). To remove genes with relatively low variability of expression, and for consistency with the previous analyses, we filtered them based on their dynamic range, computed on cRPKM after adding a pseudocount of 0.01 (see “Linear models, variance decomposition, and SVG and TVG definition”). To estimate the proportion of expression variation across organs and donors, we built a linear model for each individual gene that describes its expression as the sum of the organ and donor factors and a residual term (see “Linear models, variance decomposition, and SVG and TVG definition”). The relationship between the relative variation across donors and organs is shown in Additional file 1: Figure S10B.

## Additional files

Additional file 1
**Figures S1–S13.** File with all supplementary figures, from S1 to S13. (PDF 871 kb)

Additional file 2
**Table S1.** Intersection between gene sets identified by variance decomposition and dynamic range. (PDF 14 kb)

Additional file 3
**Table S2–S7.** Spreadsheet with multiple tables, from S2 to S7. **Table S2.** List of genome assemblies and annotation files used in the Barbosa-Morais et al. study. **Table S3.** List of 6283 protein-coding orthologs in the seven species studied here. **Table S4.** Several attributes for the 6283 orthologs. This table includes for each of the 6283 orthologs (rows), the following attributes (as columns): col 1: human gene ID (Ensembl), col 2: absolute sum of squares across organs, col 3: absolute sum of squares across species, col 4: absolute residual variation, col 5: proportion of variation across organs, col 6: proportion of variation across species, col 7: category assigned based on the proportions of variation across organs and species (see “Methods” for details), col 8: dynamic range, col 9: constrained class based on dynamic range. The final gene sets can be obtained by requiring the value in col 9 to be “unconstrained”. **Table S5.** List of genes identified by projection score. **Table S6.** List of the mouse ENCODE ChIP-seq datasets used here. **Table S7.** List of GTEx sample IDs used in the variance decomposition. (XLSX 777 kb)
