# Supplementary material for: Gene-specific patterns of expression variation across organs and species
Source: Genome Biol. 2016 Jul 8;17:151. doi: 10.1186/s13059-016-1008-y (PMC4937605; doi:10.1186/s13059-016-1008-y)

Figure S1. Modularity analysis for gene expression correlation networks derived from 2 different sets of organs. The modularity is provided as a function of the network density (number of edges over the total number of possible edges), when the vertex type is species (dashed line) or organ (solid line), for the gene expression correlation network obtained using the 5 organs that are common between the Barbosa-Morais et al. study and the Lin et al. study (namely brain, liver, kidney, heart, testes) **(A)**, and the 5 organs with the least organ- specific genes according to the Lin et al. study **(B)**. All genes in Lin et al. data (15,106 human-mouse orthologs) were used in both.

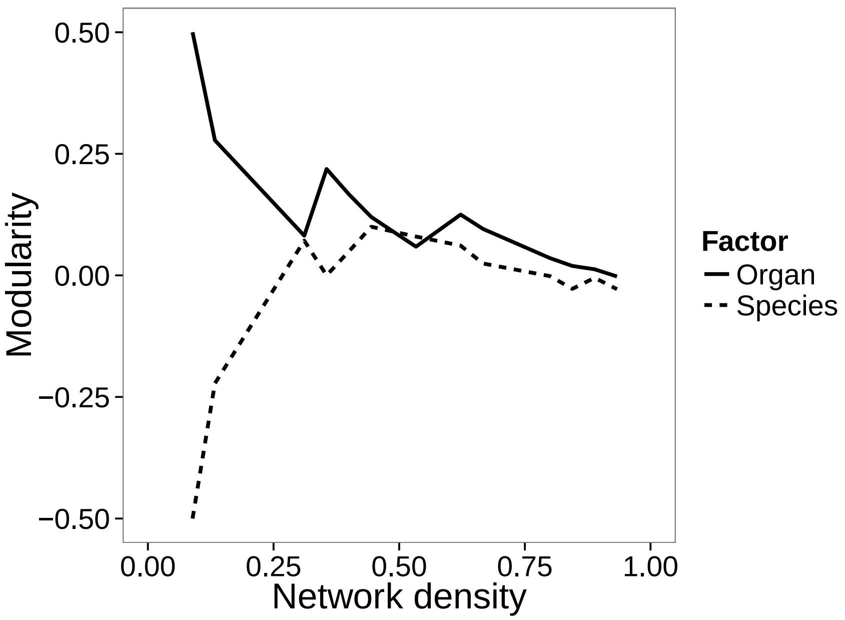

A

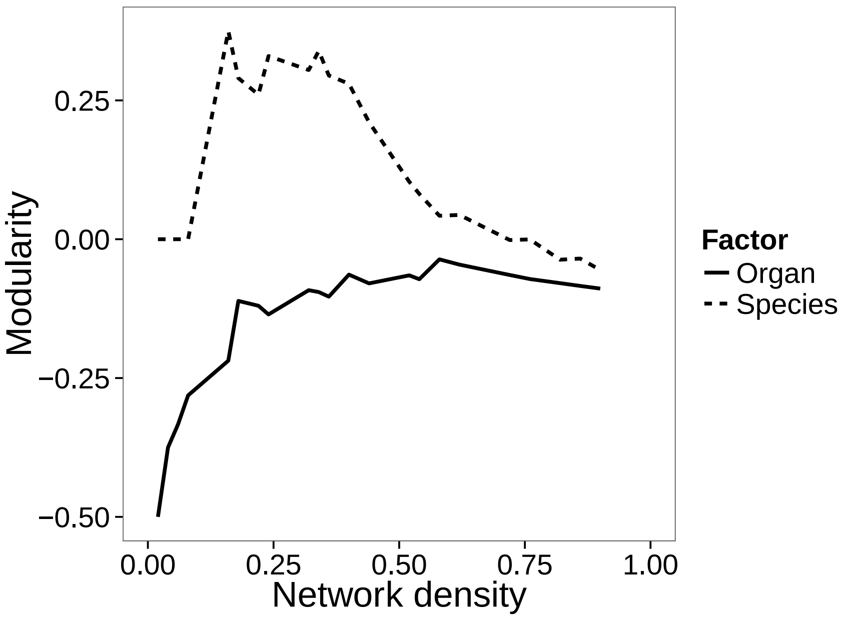

B

Figure S2. Hierarchical clustering of samples common to the Barbosa-Morais et al. and the Lin et al. studies with orthologous genes from the Barbosa-Morais et al. study (6,125 genes) (A) and human-mouse orthologs from the Lin et al. study (15,106 genes) (B).

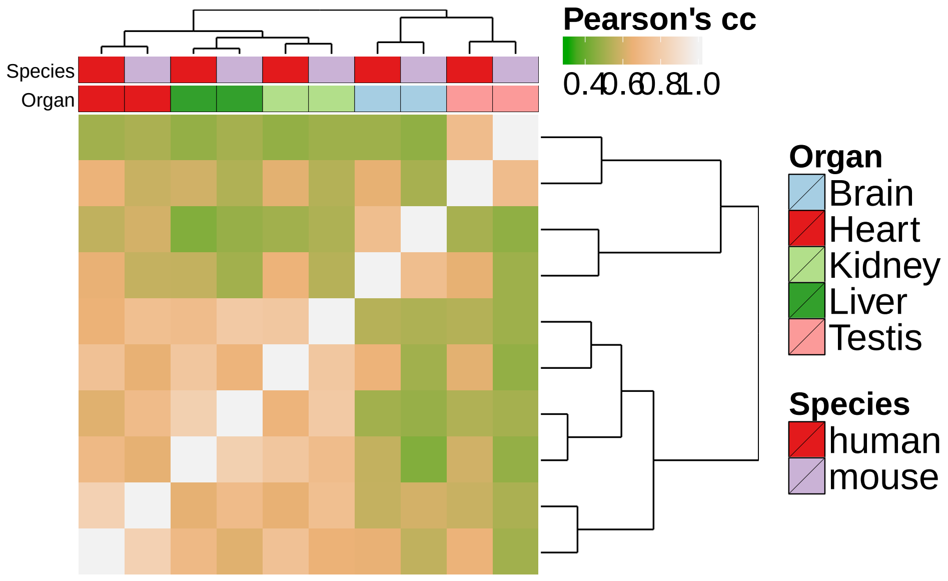

A

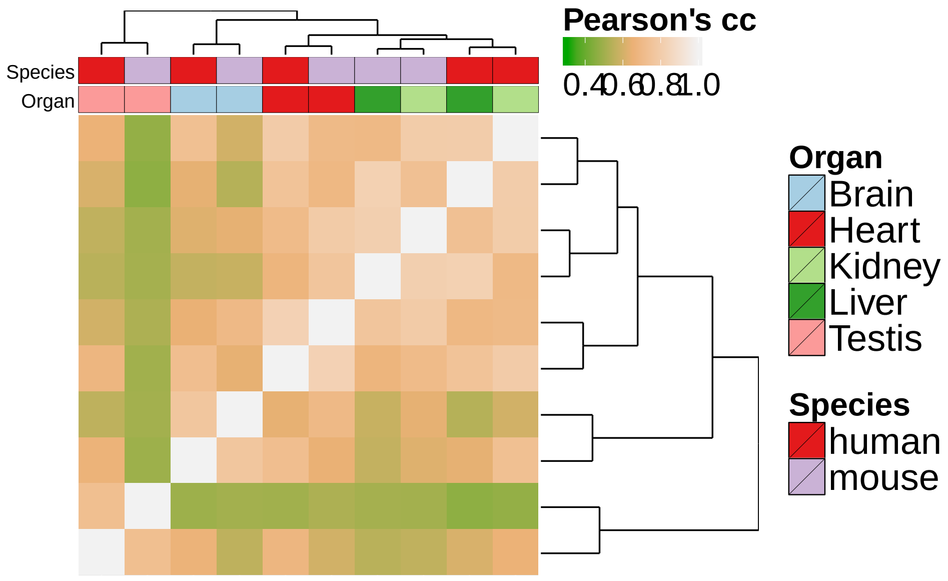

B

Figure S3. Projection score. (A) The projection score of subsets of genes is provided as a function of their variance across all samples (in fact the fraction of their variance over the maximum variance). The subset of genes with maximal projection score are the most informative with respect to PCA visualization and called projection score (proj.score or prjSc) genes. **(B)** Hierarchical clustering of samples based on the expression of the projection score genes (256 genes).

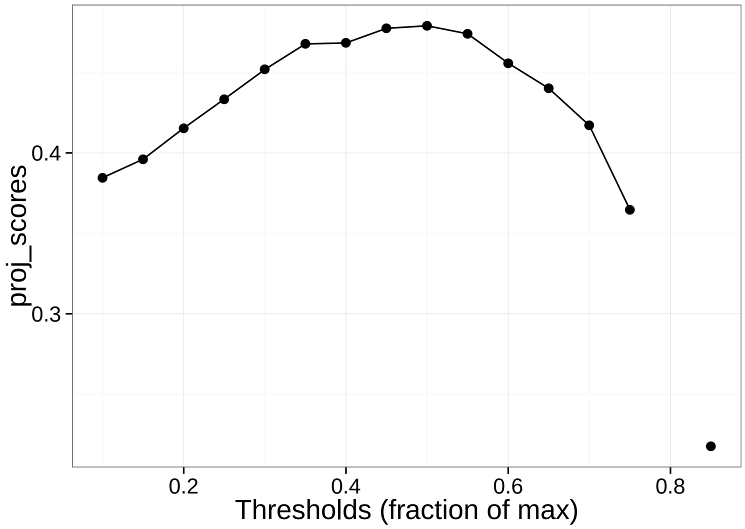

A

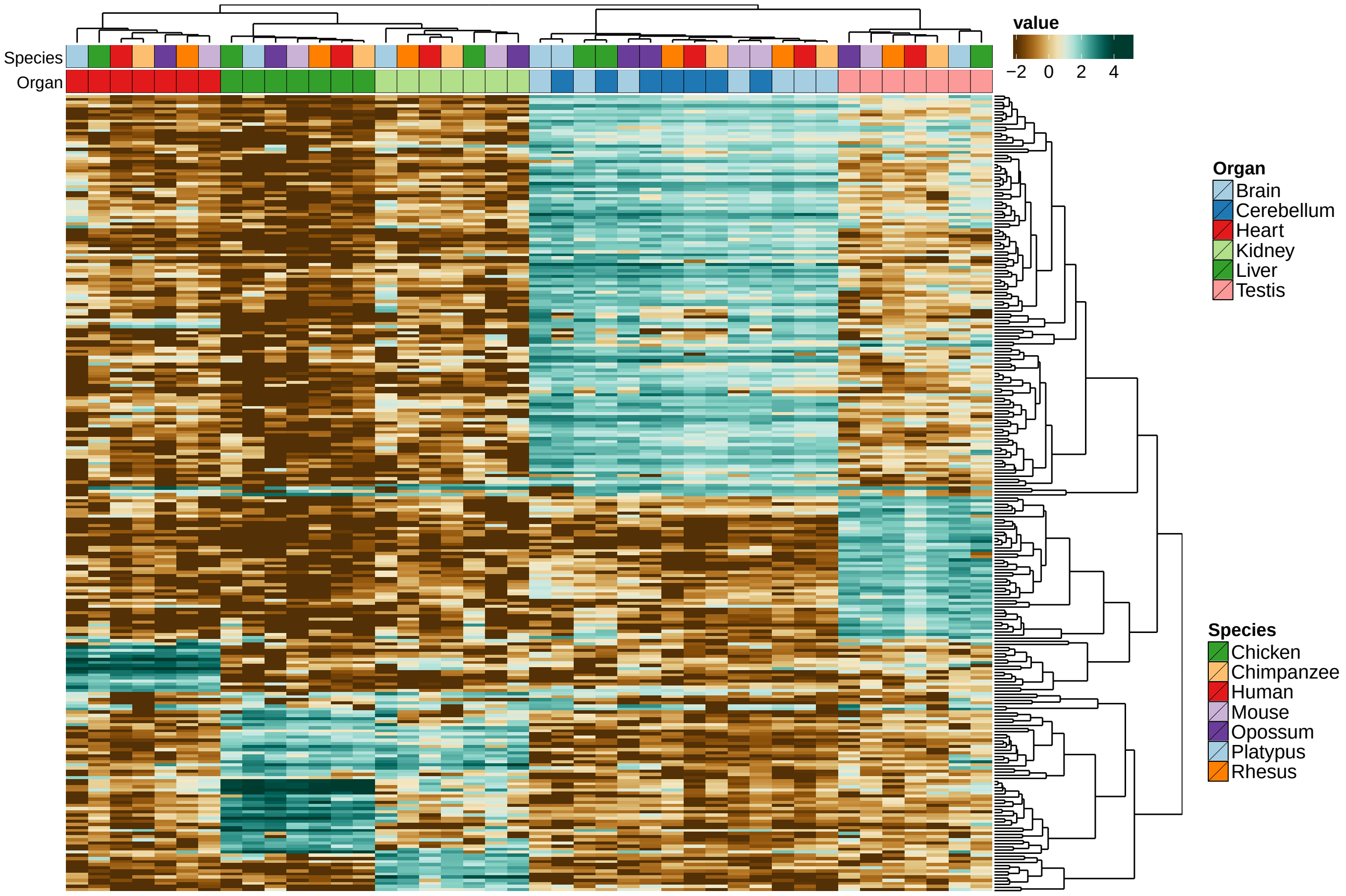

B

Figure S4. Boxplot representing the distribution of the proportion of gene expression variance across organs, except testis (light yellow) or between human and each other species (dark yellow). The trend is similar to Figure 3B even if it is computed without testis.

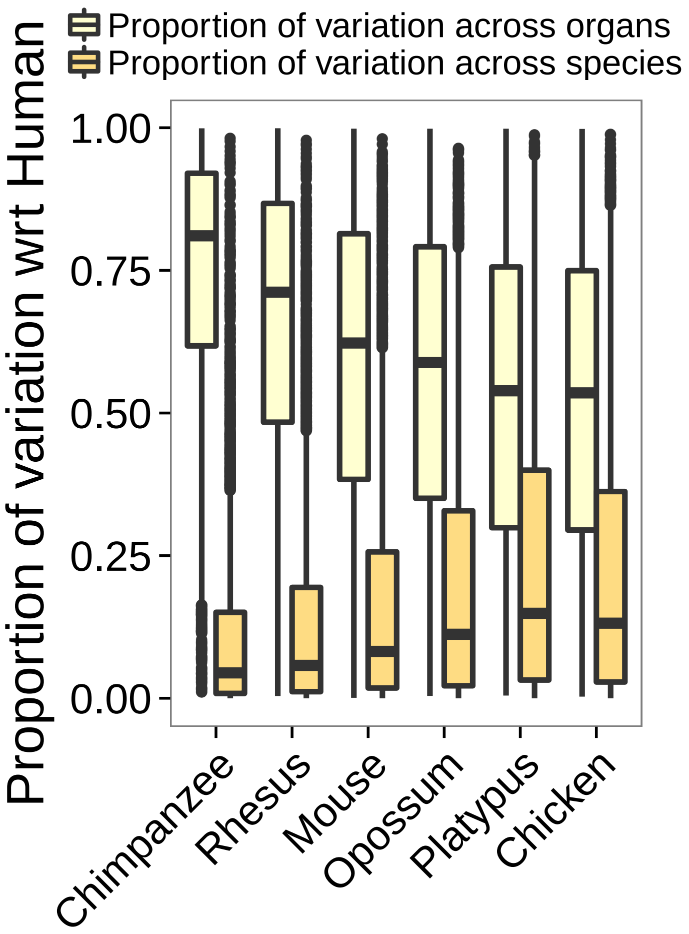

Figure S5. Absolute variance of gene expression across organs and species for different classes of genes.

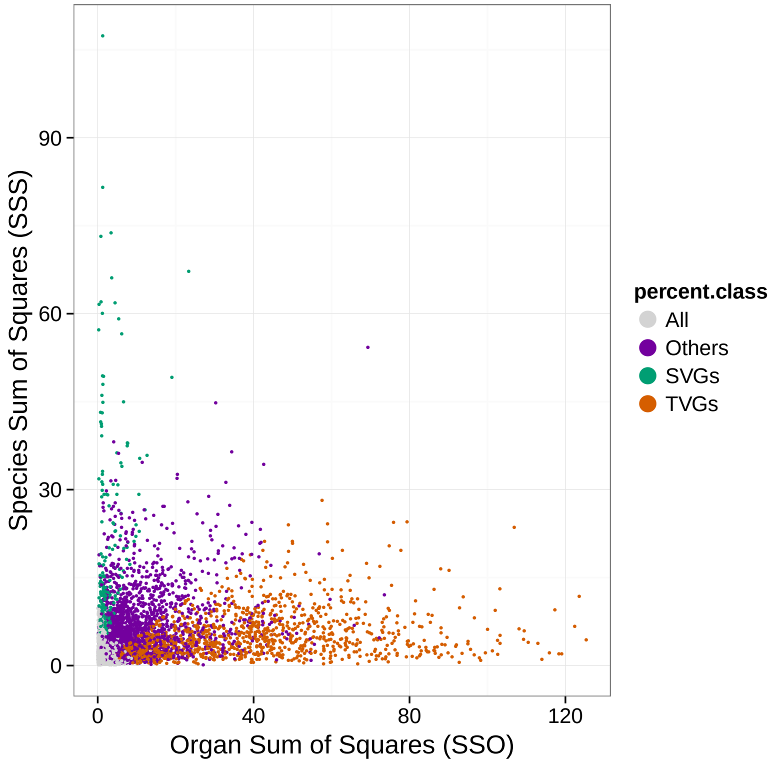

Figure S6. Hierarchical clustering based on the expression of TVGs (A) and SVGs (B).

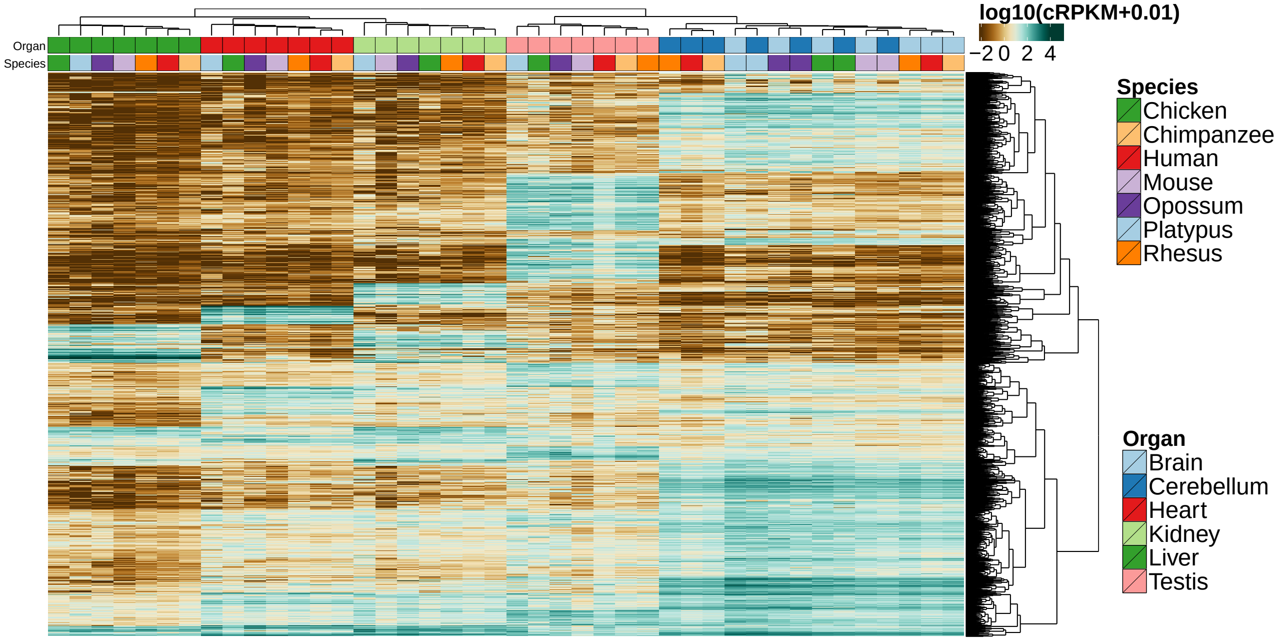

A

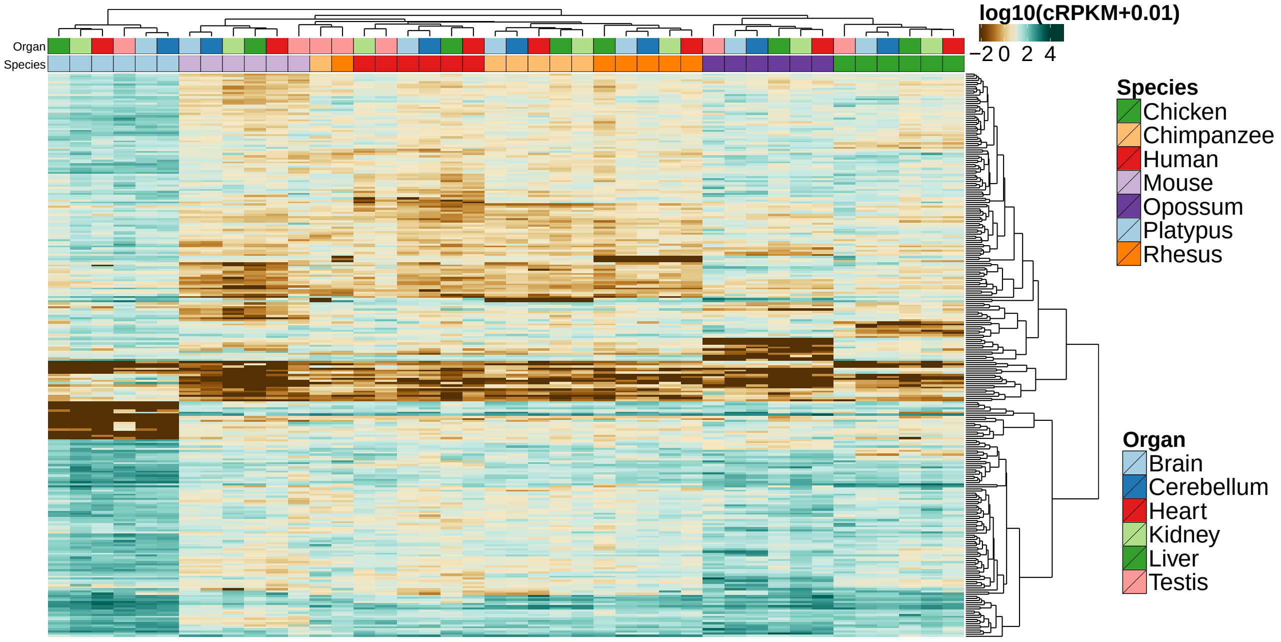

B

Figure S7. Modularity analysis of expression networks obtained from SGVs, TGVs and other genes. Modularity is given as a function of the number of edges in the network of gene expression correlations from SVGs (green), TVGs (orange) and other genes (purple) and when the vertex type is organ (circle) or species (triangle). For SVGs modularity is higher for species than for tissues. For TVGs, modularity is higher for tissues than species. Other genes show an intermediate behavior.

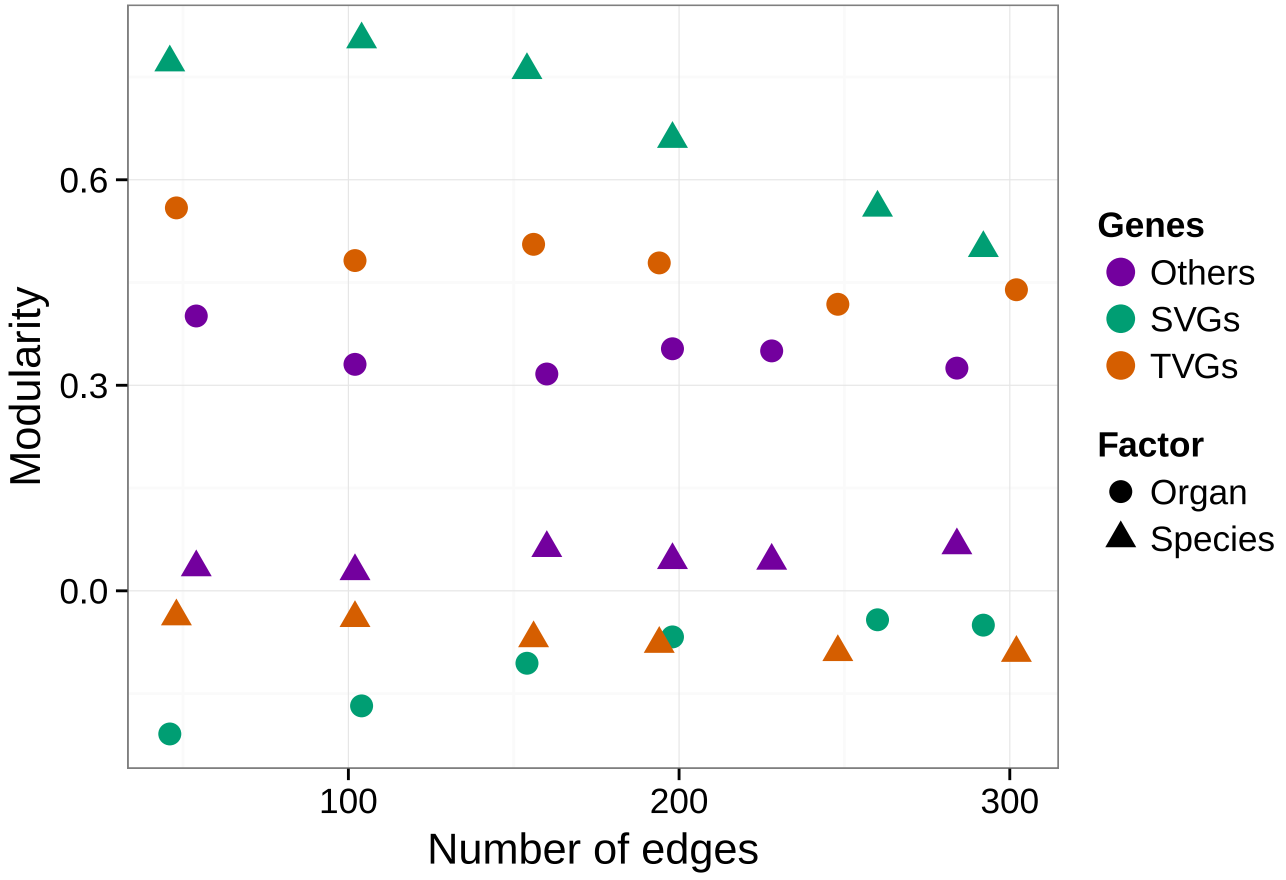

Figure S8. Gene Ontology (GO) enrichement for the top 10 significanly enriched terms in each set: SVGs (green), TVGs (brown) and other genes (purple).

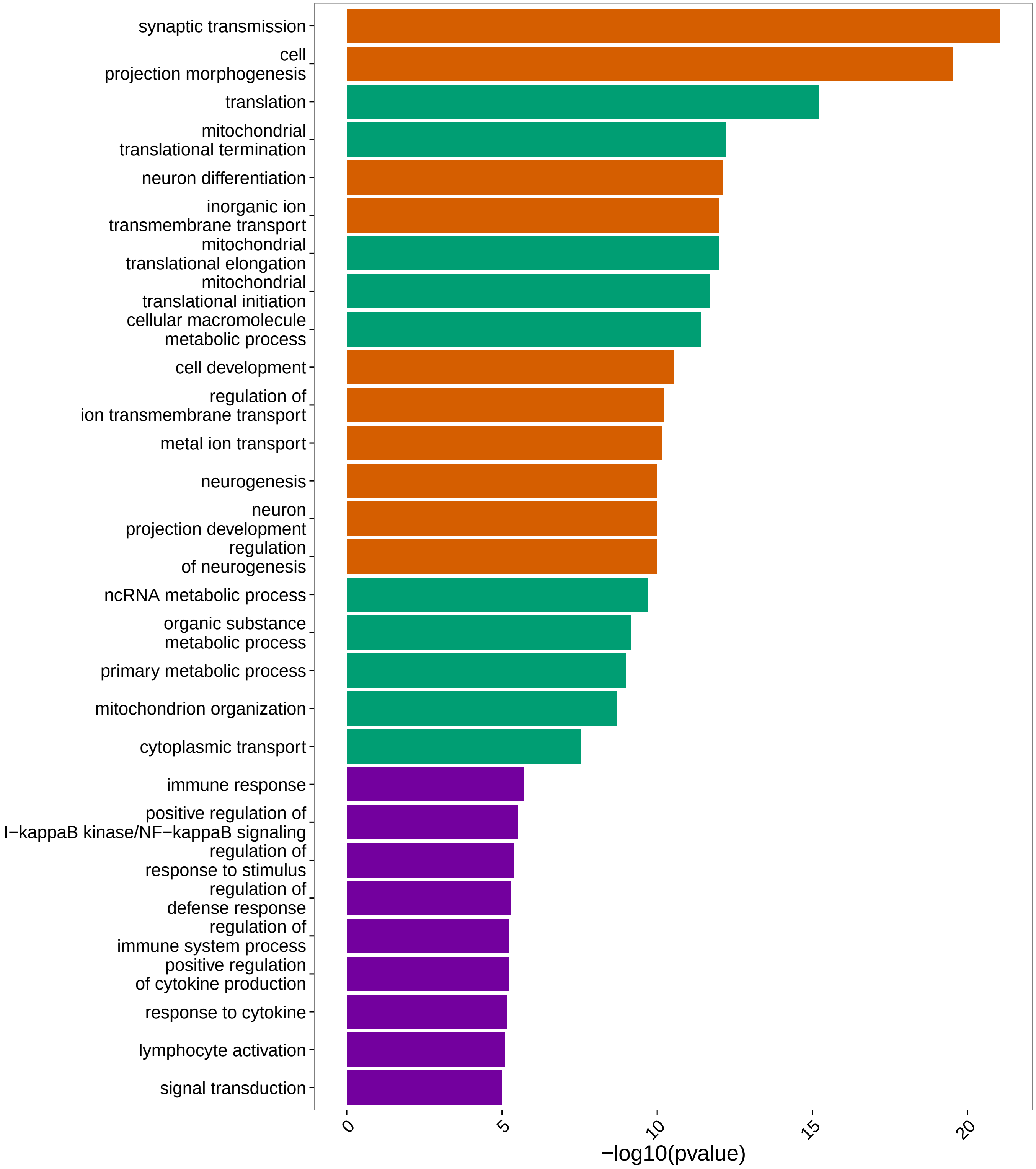

Figure S9. Profile of the histone mark H3K4me3 at the transcription start sites (TSSs). This profile is provided for tissue specific genes, as well as for sets of species specific and of other genes of the same size and expression distribution as the tissue specific genes.

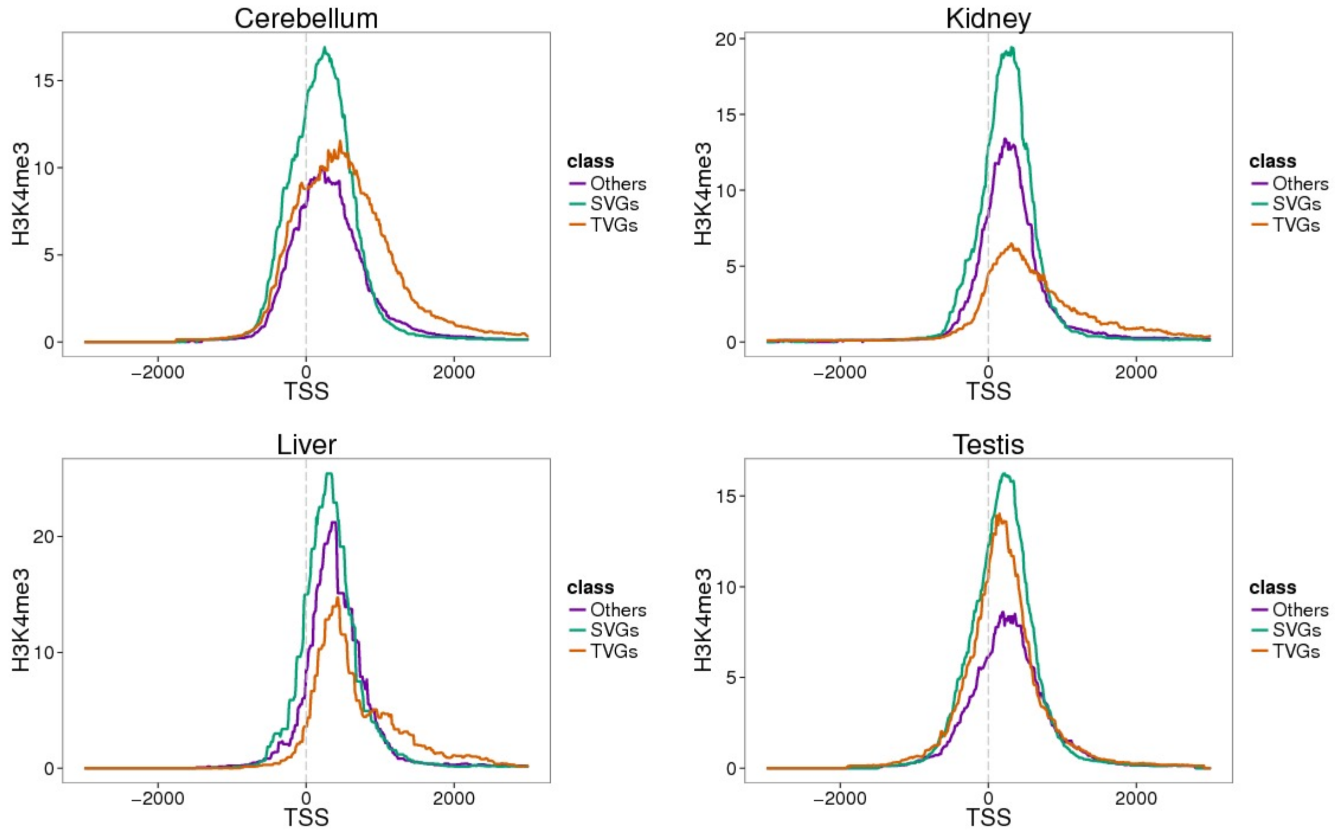

Figure S10. Analysis of relative expression variation across individuals in GTEx. A) Distribution of the proportion of variance explained by individual, as estimated in the GTEx pilot paper (Melé et al. 2015), for each class of genes. B) Proportion of expression variance explained by individuals (x axis) and by organs (y axis) for each of the 6,283 orthologous genes (dots) using linear models on GTEx samples from the four donors from which cerebellum, heart, liver, kidney and testis were available. The points with a dynamic range larger than 2 are colored according to the three classes of genes that we defined (TVGs, SVGs and Others), or in blue if they have a dyanmic range lower than 2 in the Barbosa et al. study (LowDNRBarbosa)

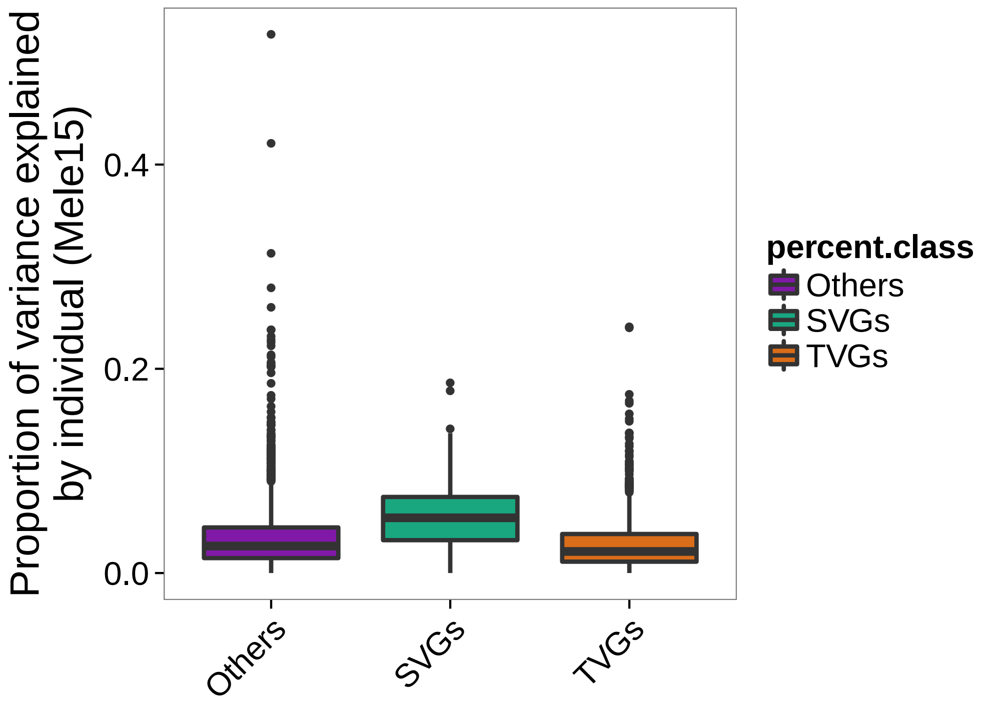

A

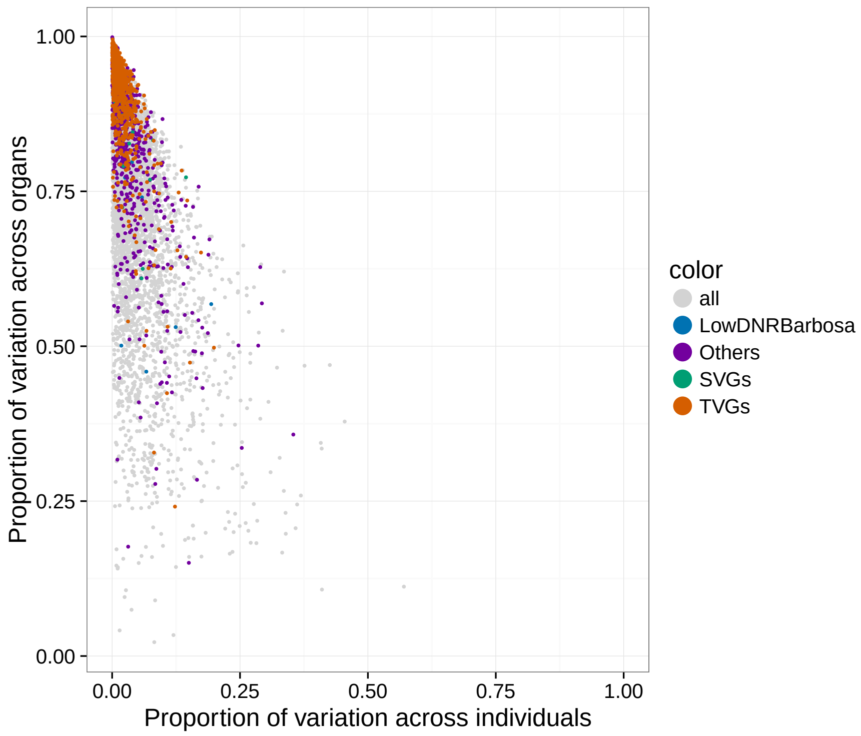

B

Figure S11. Comparison between the proportion of explained variation provided by linear models and by linear mixed models, across organs (A) and across species (B).

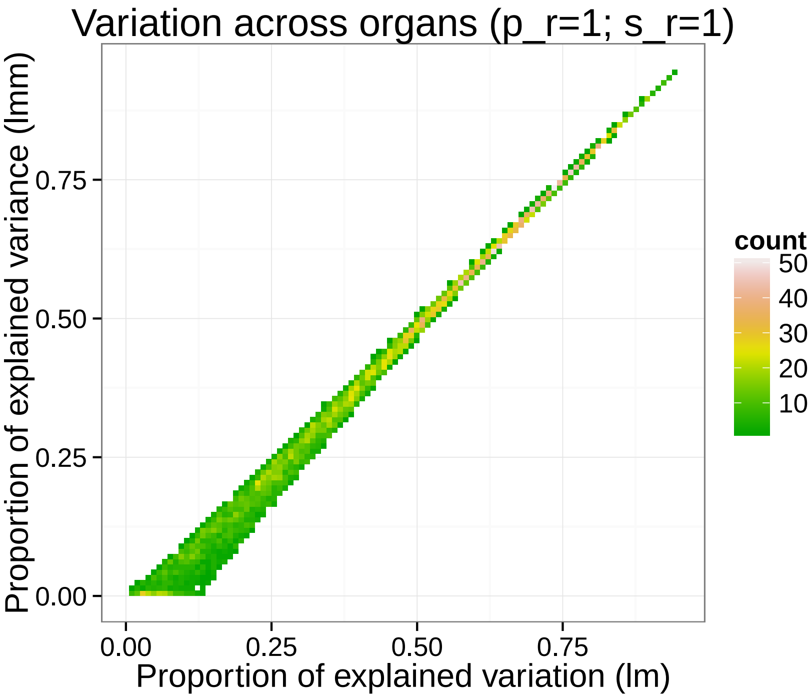

A

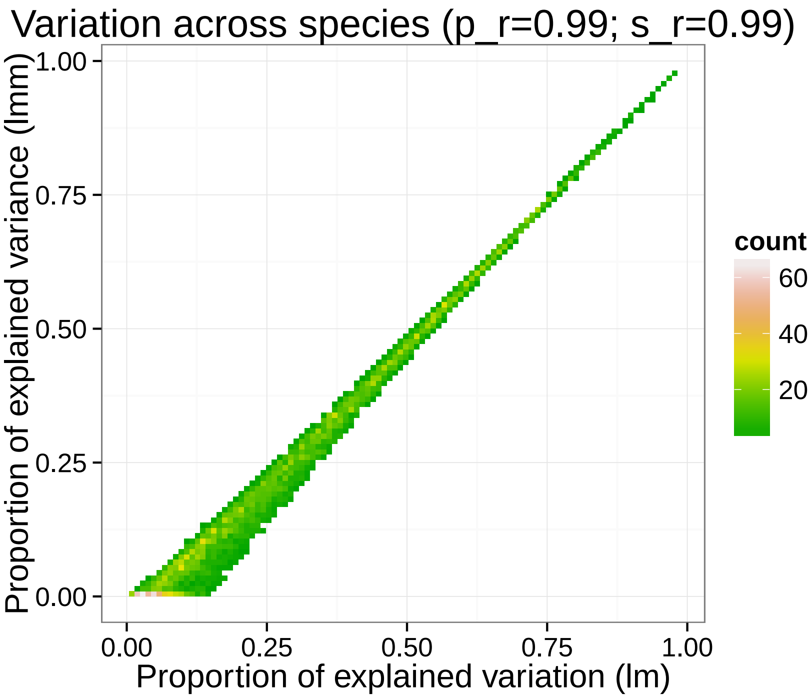

B

Figure S12. Number of genes specific to a given number of organs (color scale) at increasing thresholds of minimum number of species in which they are detected.

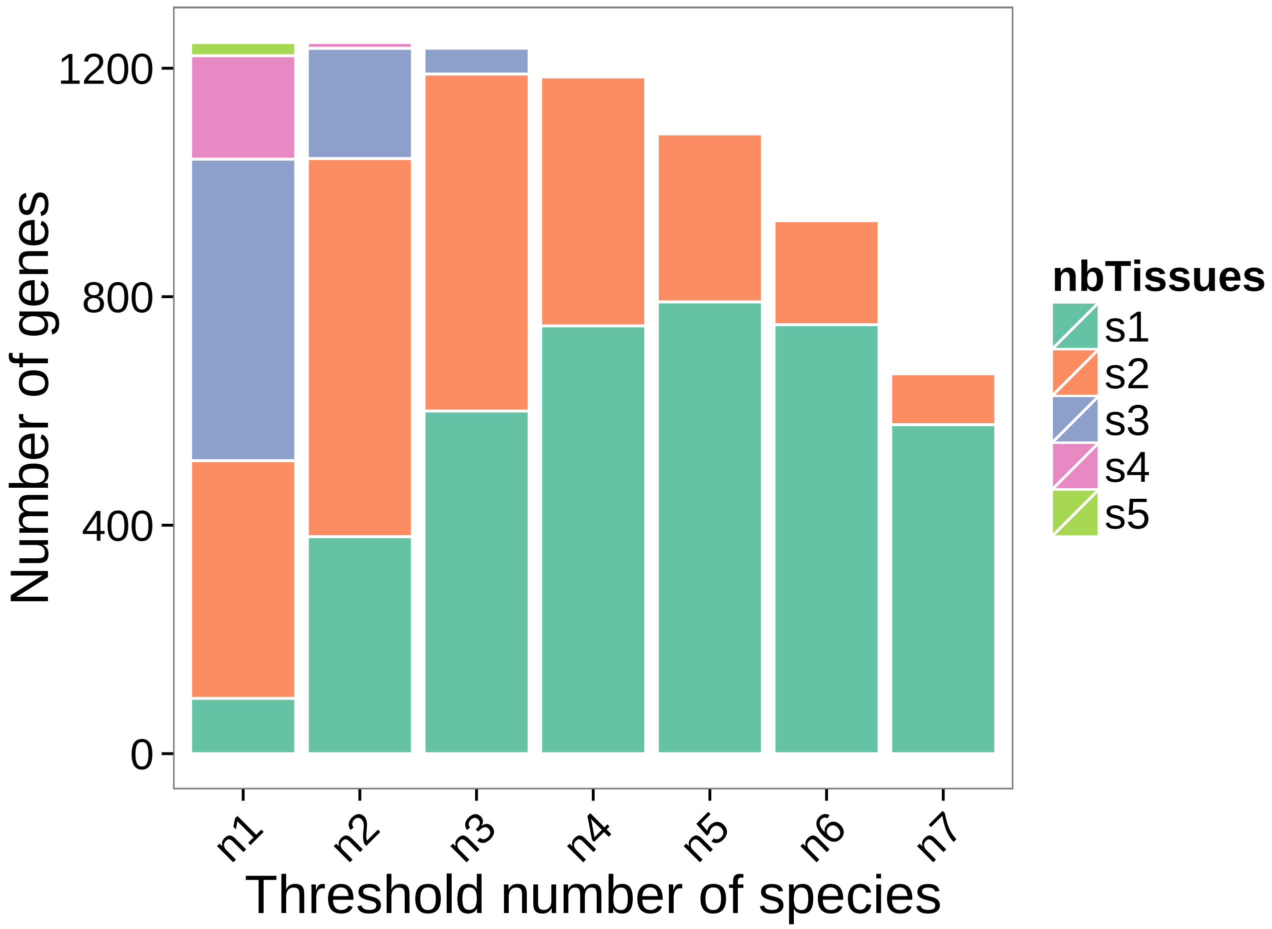

Figure S13. Distribution of the organs in which TVGs are expressed. TVGs can be expressed in different organs. For example TVGs expressed in the brain are distributed into about 200 genes expressed in the brain only, and about 200 genes expressed both in the brain and in the cerebellum.

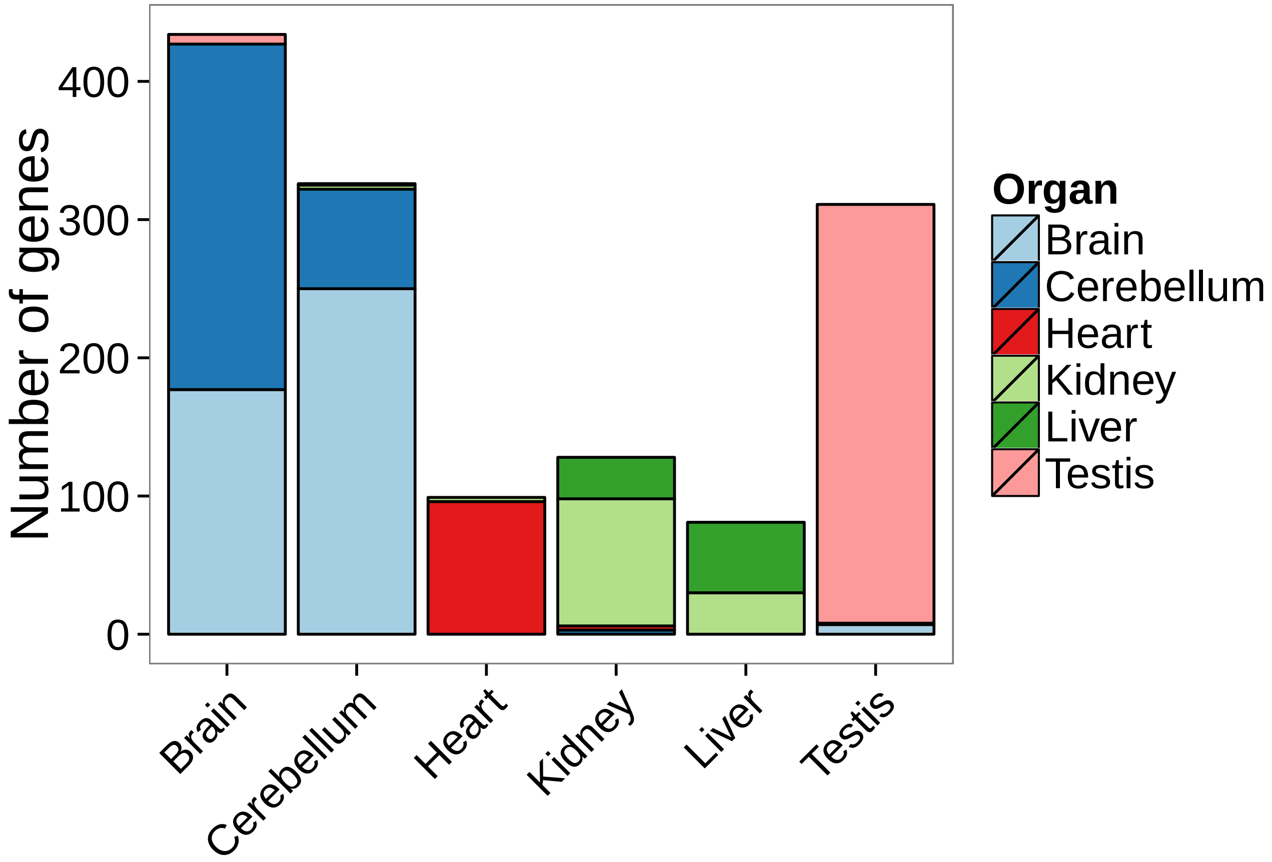

Supplement: Additional file 1 — Figures S1–S13. File with all supplementary figures, from S1 to S13. (PDF 871 kb) [file 13059_2016_1008_MOESM1_ESM.pdf]
