# Supplementary material for: Gene-specific patterns of expression variation across organs and species
Source: Genome Biol. 2016 Jul 8;17:151. doi: 10.1186/s13059-016-1008-y (PMC4937605; doi:10.1186/s13059-016-1008-y)
Supplement: Additional file 2 — Table S1. Intersection between gene sets identified by variance decomposition and dynamic range. (PDF 14 kb) [file 13059_2016_1008_MOESM2_ESM.pdf]

**Table S1. Intersection between gene sets identified by variance decomposition and dynamic range.**

| | TVGs | SVGs | Others | Total |
|---|---|---|---|---|
| Constrained | 283 | 551 | 1,827 | 2,661 |
| Unconstrained | 1,245 | 268 | 2,109 | 3,622 |
| Total | 1,528 | 819 | 3,936 | 6,283 |
